# Assessment of Factors Contributing to Infection Severity and High Levels of Drug Resistance in Clinical *Enterococcus* Isolates

**DOI:** 10.1002/jcla.70063

**Published:** 2025-05-27

**Authors:** Maryam Koleini, Ahmad Mosadegh, Farzan Madadizadeh, Hamid Heidari

**Affiliations:** ^1^ Department of Microbiology, Faculty of Medicine Shahid Sadoughi University of Medical Sciences Yazd Iran; ^2^ Center for Healthcare Data Modeling, Department of Biostatistics and Epidemiology School of Public Health, Shahid Sadoughi University of Medical Sciences Yazd Iran

**Keywords:** biofilm, *Enterococcus*, high‐level aminoglycoside resistance (HLAR), vancomycin‐resistant enterococci (VRE), virulence factors

## Abstract

**Background:**

Various factors, including virulence determinants, biofilm formation, and antimicrobial resistance, contribute to the severity of infections caused by *Enterococcus* spp.

**Methods:**

*Enterococcus* isolates were obtained from hospitalized patients in Yazd, Iran, and identified using microbiological and molecular tests. High‐level resistance, biofilm formation, and the genes encoding virulence factors and resistance were investigated following standard methods.

**Results:**

*Enterococcus faecalis* was the most prevalent species (60.7%), followed by *Enterococcus faecium* (30.4%). Linezolid was highly effective, with 94.6% of isolates being susceptible. However, more than 76% of isolates exhibited resistance to rifampin, erythromycin, tetracycline, and ciprofloxacin, and 94.6% were multidrug‐resistant (MDR). Additionally, 39.3% of the isolates were vancomycin‐resistant enterococci (VRE) with a MIC > 32 μg/mL, and the *vanA* gene was detected in 35.7% of the isolates. High‐level resistance to gentamicin and streptomycin was seen in 60.7% and 50% of the isolates, respectively. The most prevalent aminoglycoside resistance gene was *aph*(*3*′)*‐IIIa* (62.5%) followed by *ant*(*6*′)*‐Ia* (58.9%), and *aac*(*6*′)*‐Ie‐aph*(*2*″)*‐Ia* (50%). The *ant*(*3*″)*‐Ia* was found in only one isolate. Most of the isolates (87.5%) were biofilm producers, and the distribution of virulence‐encoding genes was as follows: *gelE* (66.1%), *efaA* (57.1%), *asa1* (51.8%), *esp* (25%), *cylA* (19.6%), and *hyl* (8.9%). Furthermore, the *ace* gene was present in 79.4% of *E. faecalis* isolates, while the *fnm* and *acm* genes were found in 76.5% and 23.5% of *E. faecium* isolates, respectively.

**Conclusion:**

The study highlights the significant role of notable drug resistance and the widespread presence of virulence traits in the development of enterococcal infections.

## Graphical abstract

*E. faecalis* was the predominant species (60.7%). Moreover, 39.3% of the isolates were identified as VRE with an MIC > 32 μg/mL, and the *vanA* gene was detected in 35.7% of the isolates. High‐level resistance to gentamicin and streptomycin was observed in 60.7% and 50% of the isolates, respectively. The most prevalent aminoglycoside resistance gene was *aph*(*3*′)*‐IIIa*, found in 62.5% of the isolates. Additionally, most of the isolates (87.5%) were biofilm producers, and the predominant virulence‐encoding gene was *gelE*, detected in 66.1% of the isolates.

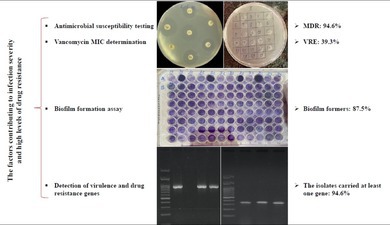

## 1 Introduction

Enterococci, particularly *Enterococcus faecalis* and *Enterococcus faecium* , are recognized as significant contributors to healthcare‐associated infections [1]. These infections commonly include urinary tract infections (UTIs), bacteremia, endocarditis, meningitis, and soft tissue infections [2].

Various virulence determinants play a role in the pathogenesis of enterococci, enabling the bacteria to survive under harsh conditions. Certain virulence factors, such as cytolysin (Cyl), gelatinase (GelE), and aggregation substance (Asa1), enhance the severity of infections and are associated with bacteremia and endocarditis. Other virulence factors, including enterococcal surface protein (Esp) and *E. faecalis* antigen A (EfaA), act as surface adhesins [3, 4]. Hyaluronidase (Hyl) and collagen adhesin (Ace) promote bacterial adherence and colonization in hospitalized patients [1]. Furthermore, specific virulence factors unique to *Enterococcus faecium* , such as collagen adhesin (Acm), a surface‐exposed adhesin (EcbA), and fibronectin‐binding protein (Fnm), facilitate the bacterium's binding to collagen and fibronectin, leading to various infections [5, 6].

The increasing significance of *Enterococcus* spp. in causing infections among hospitalized patients is attributed to their resistance to many antimicrobial agents, including β‐lactams, macrolides, fluoroquinolones, aminoglycosides, and glycopeptides such as vancomycin [1]. Resistance to vancomycin is encoded by the *van* gene clusters [7]. The expression of the *vanA* and *vanB* genes leads to the alteration of D‐alanine: D‐alanine to D‐alanine: D‐lactate, which has a lower affinity for vancomycin [8]. Resistance to aminoglycoside antibiotics, such as streptomycin and gentamicin, is mediated by aminoglycoside‐modifying enzymes (AMEs) [9]. One of the most common AME genes reported in enterococci is *aac*(*6*′)*‐Ie‐aph*(*2*″)*‐Ia*. This gene encodes an enzyme with dual functions, AAC(6′)‐APH(2″), which results in high‐level gentamicin resistance [10]. The *aph*(*2*′′)*‐Ib*, *aph*(*2*′′)*‐Ic*, and *aph*(*2*′′)*‐Id* genes also encode resistance to gentamicin. High‐level streptomycin resistance in enterococci can be enzymatic, resulting from the production of ANT(6′)‐Ia or ANT(3′′)‐Ia [11]. Genes such as *aph*(*3*′)*‐IIIa* and *ant*(*4*′)*‐Ia* can also confer resistance to several aminoglycosides [12]. One treatment for serious enterococcal infections involves the combination of a cell wall‐active agent (such as β‐lactams) with an aminoglycoside antibiotic, which produces a synergistic effect. The combined effect of two antimicrobial agents surpasses the sum of their individual actions, particularly in the case of aminoglycosides and cell‐wall‐active agents. However, if *Enterococcus* exhibits high‐level resistance to aminoglycosides, the combination therapy is likely to fail [13].

Biofilm formation is another factor contributing to the adhesion and spread of enterococci. Bacteria that form biofilms account for over 80% of bacterial infections [14]. Furthermore, biofilm formation by enterococcal species complicates treatment, as the biofilm shields bacteria from the immune response and antimicrobial effects [15].

The presence of multiple virulence factors, the ability to form biofilms, and resistance to a broad spectrum of antimicrobial agents, particularly aminoglycosides and glycopeptides, in enterococci contribute to persistent and life‐threatening infections in hospitalized patients [16]. Consequently, the aim of the present study was to evaluate these characteristics in enterococcal isolates obtained from hospitalized patients in Yazd, a central region of Iran.

## 2 Materials and Methods

### 2.1 Bacterial Isolates

In this cross‐sectional study (conducted from June to December 2022), a total of 56 *Enterococcus* isolates were collected from hospitalized patients at Shahid Sadoughi Hospital in Yazd, Iran. These isolates were obtained from cases of urinary tract infection (*n* = 41), wound infection (*n* = 8), shunt infection (*n* = 3), bloodstream infection (*n* = 2), respiratory infection (*n* = 1) and pharynx (*n* = 1). Microbiological tests, including Gram staining, the catalase test, observation of colonies with a black halo on bile‐esculin agar, and growth in Brain Heart Infusion broth with 6.5% NaCl, were performed to identify the *Enterococcus* isolates [12]. The *ddl* gene, which encodes D‐Ala:D‐Ala ligase, was detected using polymerase chain reaction (PCR) with specific primers (Table 1) for the molecular identification of *E. faecalis* and *E. faecium* [7]. All other isolates were categorized as belonging to other species. This study was approved by the ethics committee of Shahid Sadoughi University of Medical Sciences (Register code: IR. SSU.MEDICINE.REC. 1401.058).

**TABLE 1:** The primers used in the study.

Gene	Primer sequence (5′‐3′)	Amplicon size (b.p)	Ref.
*ddl* ( *E. faecalis* )	F: ATCAAGTACAGTTAGTCT R: ACGATTCAAAGCTAACTG	941	[7]
*ddl* ( *E. faecium* )	F: CCAAGGCTTCTTAGAGA R: CATCGTGTAAGCTAACTTC	535	[7]
*gelE*	F: ACCCCGTATCATTGGTTT R: ACGCATTGCTTTTCCATC	419	[17]
*esp*	F: TTGCTAATGCTAGTCCACGACC R: GCGTCAACACTTGCATTGCCGAA	954	[18]
*cylA*	F: TGGATGATAGTGATAGGAAGT R: TCTACAGTAAATCTTTCGTCA	517	[17]
*hyl*	F: ACAGAAGAGCTGCAGGAAATG R: GACTGACGTCCAAGTTTCCAA	276	[19]
*asa1*	F: GGTGCCACAATCAAATTAGG R: GATTCTTCGATTGTGTTGTAAACG	380	[18]
*ace*	F: GGAATGACCGAGAACGATGGC R: GCTTGATGTTGGCCTGCTTCCG	617	[20]
*efaA*	F: CGTGAGAAAGAAATGGAGGA R: CTACTAACACGTCACGAATG	500	[20]
*acm*	F: CAGGCAGAGATATCAGCAG R: ATTCTCATTTGTAACGACTAGC	1496	[21]
*ecbA*	F: ATTGATGTGGAAACTGAGGG R: TCCTTCTGGAGCCTCTACT	1227	[21]
*fnm*	F: ATGAGCGTTCCGTCAGAAGA R: GTTGCTCCGCCAAAACAAGA	887	[21]
*vanA*	F: AATACTGTTTGGGGGTTGCTC R: CTTTTTCCGGCTCGACTTCCT	734	[12]
*vanB*	F: CATCGCCGTCCCCGAATTTCAAA R: GATGCGGAAGATACCGTGGCT	297	[12]
*aac*(*6*′)*‐Ie‐aph*(*2*″)*‐Ia*	F: CAGGAATTTATCGAAAATGGTAGAAAAG R: CACAATCGACTAAAGAGTACCAATC	369	[19]
*aph*(*2*″)*‐Ib*	F: CTTGGACGCTGAGATATATGAGCAC R: GTTTGTAGCAATTCAGAAACACCCTT	867	[19]
*aph*(*2*″)*‐Ic*	F: CCACAATGATAATGACTCAGTTCCC R: CCACAGCTTCCGATAGCAAGAG	444	[19]
*aph*(*2*″)*‐Id*	F: GTGGTTTTTACAGGAATGCCATC R: CCCTCTTCATACCAATCCATATAACC	641	[19]
*ant*(*4*′)*‐Ia*	F: CAAACTGCTAAATCGGTAGAAGCC R: GGAAAGTTGACCAGACATTACGAACT	294	[19]
*aph*(*3*′)*‐IIIa*	F: GGCTAAAATGAGAATATCACCGG R: CTTTAAAAAATCATACAGCTCGCG	523	[19]
*ant*(*6*′)*‐Ia*	F: ACTGGCTTAATCAATTTGGG R: GCCTTTCCGCCACCTCACCG	596	[19]
*ant*(*3*″)*‐Ia*	F: ACCGTAAGGCTTGATGAAACA R: GCCGACTACCTTGGTGATCTC	624	[22]

### 2.2 Antimicrobial Susceptibility Testing and Determination of Multidrug‐Resistance

All isolates were examined for their susceptibility to various antibiotics in accordance with the guidelines of the Clinical and Laboratory Standards Institute (CLSI) [23]. The discs used were penicillin (10 units), ampicillin (10 μg), teicoplanin (30 μg), ciprofloxacin (5 μg), nitrofurantoin (300 μg), rifampin (5 μg), linezolid (30 μg), chloramphenicol (30 μg), erythromycin (15 μg), and tetracycline (30 μg). To identify high‐level gentamicin resistant (HLGR) and high‐level streptomycin resistant (HLSR) isolates, gentamicin (120 μg), and streptomycin (300 μg) discs were used. The minimum inhibitory concentration (MIC) of vancomycin for the isolates was also determined using the agar dilution method, in accordance with CLSI guideline [23].

Multidrug resistance was determined according to the definition of multidrug‐resistant (MDR) bacteria. The definition of MDR refers to an isolate that is non‐susceptible (including resistant or intermediate) to at least one antibiotic in three or more antimicrobial categories [24].

### 2.3 Biofilm Formation Assay

The ability to form biofilms was evaluated using the microtiter plate assay as described previously [16]. In brief, *Enterococcus* isolates were inoculated into 5 mL of trypticase soy broth (TSB) supplemented with 0.5% glucose and incubated overnight at 37°C. A 0.5 McFarland standard concentration was then prepared in TSB, and 100 μL of these dilutions were added to each well of a flat‐bottomed polystyrene 96‐well microtiter plate. Following a 24‐h incubation at 37°C, the supernatant was discarded, and the wells were washed three times with 150 μL of normal saline solution (0.9% NaCl). Adherent biofilms were fixed using 96% ethanol. After removing the solutions, the plate was air‐dried and stained with 100 μL of 1.5% crystal violet for 20 min. The unbound stain was washed off three times with water. The dye was subsequently dissolved in 150 μL of 33% (v/v) acetic acid. The optical densities (OD) of the wells were measured at 550 nm using a microplate reader. This procedure was repeated three times for each isolate, with *S. epidermidis* ATCC 35984 and sterile broth as positive and negative controls, respectively. A cut‐off value (ODc) was established, defined as three standard deviations (SD) above the mean OD of the negative control: ODc = average OD of the negative control + (3 × SD of the negative control). The isolates were categorized into the following four groups based on the OD: non‐biofilm producer (OD < ODc); weak‐biofilm producer (ODc < OD < 2 × ODc); moderate‐biofilm producer (2 × ODc < OD < 4 × ODc); strong‐biofilm producer (OD > 4 × ODc).

### 2.4 DNA Extraction and Molecular Analysis

Genomic DNA was extracted from freshly grown overnight colonies using a previously described method [9]. The genes analyzed in the present study were virulence genes (*gelE*, *esp*, *cylA*, *hyl*, *asa1*, *ace*, *efaA*, *acm*, *ecbA* and *fnm*), glycopeptide resistance genes (*vanA* and *vanB*) and aminoglycoside resistance genes (*aac*(*6 ´*)*‐Ie‐aph*(*2 ˝*)*‐Ia*, *aph*(*2 ˝*)*‐Ib*, *aph*(*2 ˝*)*‐Ic*, *aph*(*2 ˝*)*‐Id*, *ant*(*4 ´*)*‐Ia*, *aph*(*3 ´*)*‐IIIa*, *ant*(*6*′)*‐Ia*, and *ant*(*3 ˝*)*‐Ia*) (Table 1) [12, 17, 18, 19, 20, 21, 22].

### 2.5 Statistical Analysis

Frequency (percentage) was used for description. The distribution of the genes and factors across different groups of isolates, including enterococcal species, biofilm formers versus non‐biofilm formers, and resistant versus susceptible isolates, was investigated using Chi‐square and Fisher's Exact Tests. All statistical analyses were conducted using SPSS software version 26. A *p*‐value of < 0.05 was considered statistically significant.

## 3 Results

During the study, 34 isolates (60.7%) of *E. faecalis* and 17 isolates (30.4%) of *E. faecium* were identified. Additionally, five isolates (8.9%) were non‐*faecalis*/non‐*faecium* and were considered other species.

### 3.1 Antimicrobial Resistance Patterns

As shown in Table 2, the highest resistance prevalence was observed to rifampin (85.7%), erythromycin (80.4%), tetracycline (78.6%), and ciprofloxacin (76.8%), and the most efficient antibiotic was linezolid with 94.6% susceptibility. Thirty‐four (60.7%) and 28 (50%) isolates were HLGR and HLSR, respectively. Vancomycin resistance was observed in 22 (39.3%) isolates, with MIC > 32 μg/mL (Table 3). Resistance to vancomycin and non‐susceptibility to teicoplanin, ampicillin (*p* = 0.001), and penicillin (*p* = 0.003) in *E. faecium* were significantly higher than in *E. faecalis* isolates. Additionally, non‐susceptibility to tetracycline in *E. faecalis* was significantly higher than in *E. faecium* isolates (*p* = 0.01). Furthermore, 53 isolates (94.6%) were MDR.

**TABLE 2:** Antimicrobial resistance patterns of the isolates.

Class of antimicrobial agents	Antibiotic	*N* (%)		
		S	I	R
Oxazolidinone	Linezolid	53 (94.6)	1 (1.8)	2 (3.6)
Aminoglycoside	Streptomycin	28 (50)	—	28 (50)^a^
	Gentamicin	22 (39.3)	—	34 (60.7)^a^
Ansamycin	Rifampin	5 (8.9)	3 (5.4)	48 (85.7)
Nitrofuran	Nitrofurantoin	49 (87.5)	1 (1.8)	6 (10.7)
Phenicol	Chloramphenicol	42 (75)	5 (8.9)	9 (16.1)
Quinolone	Ciprofloxacin	3 (5.4)	10 (17.8)	43 (76.8)
Tetracycline	Tetracycline	11 (19.6)	1 (1.8)	44 (78.6)
Macrolide	Erythromycin	2 (3.5)	9 (16.1)	45 (80.4)
Penicillin	Penicillin	23 (41.1)	—	33 (58.9)
	Ampicillin	31 (55.4)	—	25 (44.6)
Glycopeptide	Vancomycin	34 (60.7)	—	22 (39.3)
	Teicoplanin	33 (58.9)	2 (3.6)	21 (37.5)

Abbreviations: I, intermediate; R, resistant; S, susceptible.

^a^ High‐level resistant.

**TABLE 3:** MIC of vancomycin against the isolates.

MIC (μg/mL)	*N* (%)				
	≤ 2	4	8	16	> 32
*E. faecalis* (34)	23 (67.6)	5 (14.7)	—	—	6 (17.6)
*E. faecium* (17)	2 (11.8)	1 (5.9)	—	—	14 (82.4)
Other species (5)	3 (60)	0	—	—	2 (40)
Total (56)	28 (50)	6 (10.7)	—	—	22 (39.3)

Abbreviation: MIC, minimum inhibitory concentration.

### 3.2 Biofilm Formation

**FIGURE 1:**
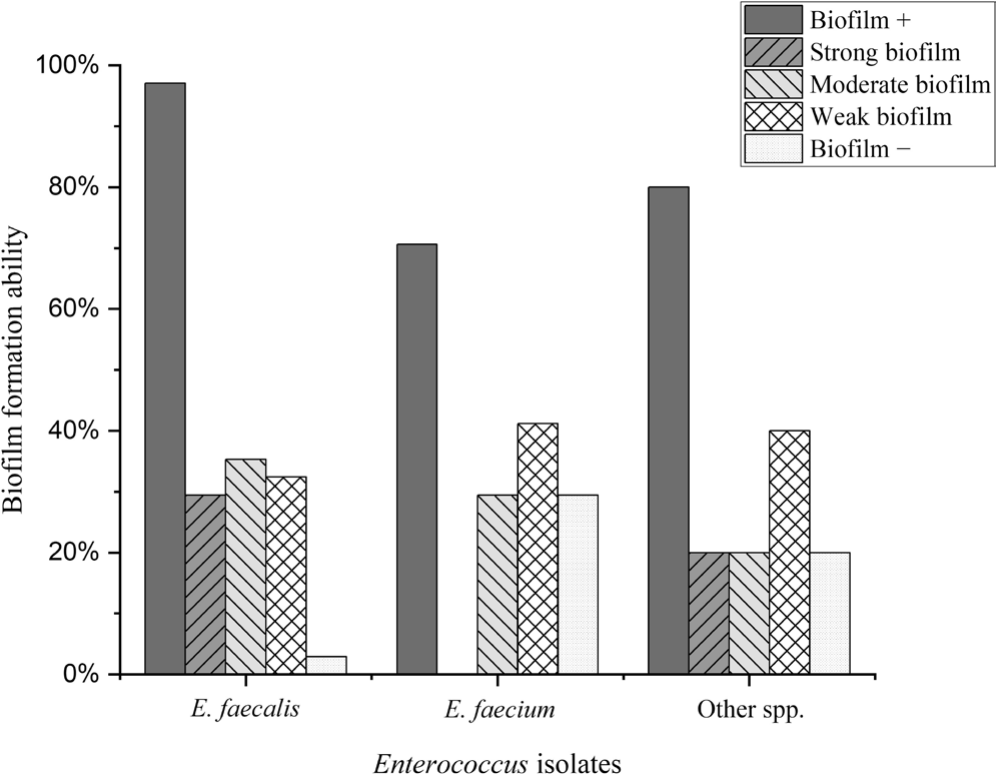
Biofilm formation in clinical isolates of *E. faecalis* (97.1%), *E. faecium* (70.6%) and other spp. (80%).

Most of the isolates formed biofilm (87.5%), and 11 (19.7%), 18 (32.1%) and 20 (35.7%) isolates developed strong, moderate, and weak biofilm, respectively. Biofilm formation in *E. faecalis* isolates was significantly higher than in *E. faecium* isolates (*p* = 0.012) (Figure 1).

### 3.3 Prevalence of Virulence‐Encoding Genes

The *gelE* was the most commonly detected gene (66.1%), followed by *efaA* (57.1%), *asa1* (51.8%), *esp* (25%), *cylA* (19.6%), and *hyl* (8.9%). The *ace* was also found in 79.4% of *E. faecalis* isolates, and the prevalence of *fnm* and *acm* genes among *E. faecium* isolates was 76.5% and 23.5%, respectively. None of the *E. faecium* isolates carried *ecbA* (Table 4). Moreover, the frequency of *gelE* and *asa1* genes among *E. faecalis* was significantly higher than in *E. faecium* isolates (*p* = 0.001).

**TABLE 4:** Prevalence of virulence‐encoding genes among the isolates.

*N* (%)										
Isolate	*gelE*	*efaA*	*asa1*	*ace*	*esp*	*fnm*	*cylA*	*hyl*	*acm*	*ecbA*
*E. faecalis* 34 (60.7)	29 (85.3)	31 (91.2)	27 (79.4)	27 (79.4)	10 (29.4)	—	10 (29.4)	1 (2.9)	—	—
*E. faecium* 17 (30.4)	7 (41.2)	0	1 (5.9)	—	4 (23.5)	13 (76.5)	1 (5.9)	3 (17.6)	4 (23.5)	0
Other species 5 (8.9)	1 (20)	1 (20)	1 (20)	—	0	—	0	1 (20)	—	—
Total 56 (100)	37 (66.1)	32 (57.1)	29 (51.8)	27 (48.2)	14 (25)	13 (23.2)	11 (19.6)	5 (8.9)	4 (7.1)	0

The ability to develop biofilm in the isolates possessing the *ace*, *asa1*, and *efaA* virulence genes was significant (*p* = 0.011, 0.004 and 0.001). In other words, all isolates positive for these virulence genes were significantly capable of producing biofilm (Table 5).

**TABLE 5:** Relation between the presence of virulence genes and biofilm formation.

Isolate (*N*)	*N* (%)			*p*
	*ace*	*asa1*	*efaA*	
Biofilm former (49)	27 (55.1)	29 (59.2)	32 (65.3)	< 0.05
Non‐biofilm former (7)	0	0	0	
Total (56)	27 (48.2)	29 (51.8)	32 (57.1)	

### 3.4 Prevalence of Antimicrobial Resistance Genes

The *vanA* was detected in 20 (35.7%) isolates, and all isolates were negative for *vanB* gene amplification. The prevalence of *vanA* gene among *E. faecium* isolates was significantly higher than in *E. faecalis* isolates (*p* = 0.001) (Table 6). This gene was also significantly more frequent in vancomycin‐resistant and teicoplanin non‐susceptible isolates compared with susceptible isolates (*p* = 0.001) (Tables 7 and 8).

**TABLE 6:** Prevalence of *vanA* gene among *E. faecalis* and *E. faecium* isolates.

Isolate (*N*)	*vanA* N (%)		*p*
	Positive	Negative	
*E. faecalis* (34)	7 (20.6)	27 (79.4)	0.001
*E. faecium* (17)	12 (70.6)	5 (29.4)	
Total (51)	19 (37.3)	32 (67.2)	

**TABLE 7:** Prevalence of *vanA* gene among vancomycin‐resistant and susceptible isolates.

Isolates (*N*)	*vanA* N (%)		*p*
	Positive	Negative	
Vancomycin susceptible (34)	3 (8.8)	31 (91.2)	0.001
Vancomycin resistant (22)	17 (77.3)	5 (22.7)	
Total (56)	20 (35.7)	36 (64.3)	

**TABLE 8:** Prevalence of *vanA* gene among teicoplanin‐susceptible and non‐susceptible isolates.

Isolate (*N*)	*vanA* N (%)		*p*
	Positive	Negative	
Teicoplanin susceptible (33)	3 (9.1)	30 (90.9)	0.001
Teicoplanin non‐susceptible (23)	17 (73.9)	6 (26.1)	
Total (56)	20 (35.7)	36 (64.3)	

The *aac*(*6*′)*‐Ie‐aph*(*2*″)*‐Ia*, *aph*(*3*′)*‐IIIa*, and *ant*(*6*′)*‐Ia* resistance genes were found in 28 (50%), 35 (62.5%), and 33 (58.9%) isolates, respectively. Only one (1.8%) isolate harbored *ant*(*3*″)*‐Ia* (Accession number: OR449308). Other AMEs‐encoding genes, *ant*(*4*′)*‐Ia*, *aph*(*2*″)*‐Ib*, *aph*(*2*″)*‐Ic*, and *aph*(*2*″)*‐Id* were not found in the isolates. The occurrence of *aac*(*6*′)*‐Ie‐aph*(*2*″)*‐Ia* among HLGR isolates was significantly higher than in non‐HLGR isolates (*p* = 0.001) (Table 9). Also, the *aac*(*6*′)*‐Ie‐aph*(*2*″)*‐Ia*, *aph*(*3*′)*‐IIIa*, and *ant*(*6*′)*‐Ia* genes were significantly more frequent in HLSR compared to non‐HLSR isolates (*p* = 0.001) (Table 10).

**TABLE 9:** Prevalence of *aac*(*6*′)*‐Ie‐aph*(*2*″)*‐Ia* gene among HLGR and non‐HLGR isolates.

Isolates (*N*)	*aac*(*6*′)*‐Ie‐aph*(*2*″)*‐Ia* N (%)		*p*
	Positive	Negative	
HLGR (34)	23 (67.6)	11 (32.4)	0.001
Non‐HLGR (22)	5 (22.7)	17 (77.3)	
Total (56)	28 (50)	28 (50)	

**TABLE 10:** Prevalence of *aac*(*6*′)*‐Ie‐aph*(*2*″)*‐Ia, aph*(*3*′)*‐IIIa* and *ant*(*6*′)*‐Ia* gene among HLSR and non‐HLSR isolates.

Isolates (*N*)	*N* (%)						*p*
	*aac(6′)‐Ie‐aph(2″)‐Ia*		*aph(3′)‐IIIa*		*ant(6′)‐Ia*		
	Positive	Negative	Positive	Negative	Positive	Negative	
HLSR (28)	21 (75)	7 (25)	25 (89.3)	3 (10.7)	25 (89.3)	3 (10.7)	0.001
Non‐HLSR (28)	7 (25)	21 (75)	10 (35.7)	18 (64.3)	8 (28.6)	20 (71.4)	
Total (56)	28 (50)	28 (50)	35 (62.5)	21 (37.5)	33 (58.9)	23 (41.1)	

## 4 Discussion

In the present study, 73.2% of the isolates were obtained from UTI, which is consistent with earlier studies reporting that the majority of enterococcal species are isolated from this type of infection [25, 26, 27]. The presence of several features, such as binding factors (aggregation substances, enterococcal surface proteins, collagen‐binding protein, and gelatinase), as well as the ability to develop biofilms, are significant risk factors for urinary tract infections [28].

According to our results, 60.7% of isolates were *E. faecalis* , 30.4% were *E. faecium* , and 8.9% were other *Enterococcus* species. The predominance of *E. faecalis* among the isolates analyzed in the study was similar to the results of several studies conducted in Iran (Hamadan, Tehran and Ahwaz) [25, 29, 30], Saudi Arabia, India, Mexico, and Northern European countries (Norway, Sweden, Denmark, and Iceland) [31, 32, 33, 34]. Meanwhile, *E. faecium* was the more prevalent species in reports from Taiwan, Japan, China, and South Korea [35, 36, 37, 38]. Although *E. faecalis* was more prevalent among clinical enterococci in Iran, the frequency of *E. faecium* was higher in clinical isolates from East Asian countries. Differences in the distribution of enterococcal species could be due to geographical variations or the types of specimens studied.

We found that 39.3% of the isolates were vancomycin‐resistant enterococci (VRE). According to meta‐analysis studies conducted in Iran in 2016 and 2018, the prevalence of VRE among clinical *Enterococcus* isolates in the country was 9.4% (ranging from 3.8% to 20.4%) and 14% (ranging from 2% to 48.9%), respectively [39, 40]. However, higher rates were reported in recent years [41, 42]. According to the studies conducted in Africa and Europe, resistance to vancomycin among *Enterococcus* spp. was 26.8% and up to 13%, respectively [43, 44]. The prevalence and colonization of VRE strains are influenced by various factors, including differences in treatment policies, the duration of hospitalization, infection control strategies in healthcare facilities, and the transmission of resistant strains between patients through medical devices or healthcare workers' hands [45].

Teicoplanin resistance was detected in 37.5% of isolates. A study conducted in Ahwaz, Iran, reported a higher prevalence (43.4%), which exceeds our findings [46]. In contrast, studies from Saudi Arabia, India, Spain, and China showed resistance rates of 1.2%, 14%, 10.9%, and 0%, respectively [47, 48, 49, 50]. Furthermore, our study revealed that *E. faecium* isolates exhibited significantly higher non‐susceptibility to teicoplanin compared to *E. faecalis* isolates. The *van* genes, which are responsible for this resistance, have been predominantly identified in *E. faecium* strains [51, 52].

In this study, 50% and 60.7% of the isolates were classified as HLSR and HLGR, respectively. The prevalence of HLSR clinical enterococci in previous studies conducted in Iran was 40.5% and 56.7% [53, 54]. Furthermore, the rate of high‐level resistance to streptomycin in India, Malaysia, and the Netherlands ranged from 42% to 49.3%, which is consistent with our findings [55, 56, 57]. Previous studies in Iran reported high‐level resistance to gentamicin, ranging from 44% to 53% [1, 25]. Lower rates of HLGR isolates were observed in neighboring countries, such as Saudi Arabia (20.2%) and Turkey (40%) [47, 58]. Moreover, a meta‐analysis by Shams Abadi et al. indicated that the pooled prevalence of HLGR *Enterococcus* isolates in Iran was 49.4% [58]. The notable prevalence of HLGR *Enterococcus* isolates may be attributed to their ability to endure harsh conditions and spread among hospitalized patients [59].

In our study, high rates of resistance to erythromycin (80.4%), tetracycline (78.6%), ciprofloxacin (76.8%), and rifampin (85.7%) were observed. Several reports from various regions, including Iran, Saudi Arabia, Ethiopia, and Croatia, have also documented notable tetracycline resistance, ranging from 64.9% to 92.1% [1, 26, 60, 61]. Additionally, the prevalence of erythromycin‐susceptible isolates has been consistently low in previous studies [62, 63]. Tetracycline resistance is highly prevalent in enterococci and is often mediated by the *tet* genes, which are carried on conjugative transposons. Furthermore, resistance to erythromycin may result from the extensive and widespread use of macrolides [9]. It seems that these antibiotics have lost their effectiveness against *Enterococcus* strains. In addition, high rates of resistance to ciprofloxacin and rifampin have been reported in earlier studies [16, 64, 65]. Ciprofloxacin is highly effective against Gram‐negative bacteria but exhibits limited activity against anaerobic and Gram‐positive bacteria [66]. As for rifampin, it serves as an effective antimicrobial agent against biofilm‐forming strains [67], and resistance to rifampin may present challenges in the treatment of bacterial biofilm‐associated infections.

According to our results, 94.6% of the isolates were MDR. Although most of the isolates in the present study were *E. faecalis* and *E. faecium* , notable multidrug resistance has been reported in various *Enterococcus* species in recent studies [68, 69]. Over recent years, the rising occurrence of MDR enterococci has significantly reduced the availability of effective therapeutic options [70].

In this study, 87.5% of isolates formed biofilm. Studies conducted in Iran have reported biofilm production rates ranging from 26.5% to 100% of the isolates [16, 65, 71]. Based on the studies in Egypt, Poland, Turkey, and Malaysia, this property was reported in 97.1%, 100%, 10%, and 52.6% of isolates, respectively [15, 72, 73, 74]. We found that 38 out of the 49 biofilm‐forming isolates were associated with UTI. Moreover, all the aforementioned studies from Iran [16, 65, 71] indicated that the highest frequency of *Enterococcus* isolates was associated with UTIs. Biofilms are responsible for the majority of bacterial infections, and biofilm‐forming enterococci exhibit higher resistance to antibiotics compared to their planktonic cells [14]. We observed that *E. faecalis* isolates had a significantly higher ability to develop biofilm compared to other species. Furthermore, the *gelE*, *efaA*, and *cylA* virulence genes, which are associated with biofilm production in enterococci, were more commonly found in *E. faecalis* isolates. Various studies showed the same results [75, 76, 77].

Regarding virulence traits, the *esp* and *cylA* genes were detected in 25% and 19.6% of the isolates, respectively. In contrast, these virulence factors were more prevalent in *Enterococcus* isolates reported in earlier studies conducted in Iran [1, 3, 19]. The occurrence of *esp* and *cylA* has varied across studies from Egypt, Turkey, and European countries [72, 73, 78]. Additionally, the *ace*, *asa1*, and *gelE* genes were present in 48.2%, 51.8%, and 66.1% of the isolates we examined, respectively. Regional differences in the prevalence of *ace* and *gelE* have also been observed [49, 79].

In our study, 34 out of 37 *gelE*‐positive isolates were able to produce biofilm. Seno et al.'s study indicated a significant link between the presence of gelatinase and the ability to form biofilm [80]. Additionally, all 11 *cylA*‐positive isolates were able to form biofilm. We also observed that the *cylA* gene was more commonly found in *E. faecalis* isolates, aligning with findings from previous studies [3, 78].

The frequency of *asa1* in analyzed isolates was comparable to previous findings [81, 82]. We found that 22 out of 29 *asa1*‐positive isolates and 10 out of 14 *esp*‐positive isolates were responsible for UTIs. A strong correlation has been demonstrated between the presence of the *esp* and *asa1* genes and the ability of enterococcal isolates to cause UTIs [19, 78].

Overall, the *hyl* gene was the least frequently detected among the virulence‐encoding genes, consistent with previous research [19, 49, 73]. Moreover, all *hyl*‐positive isolates were HLGR. In 2016, Heidari et al. reported a significant correlation between the presence of this gene and highlevels of gentamicin resistance [9]. This could be due to the simultaneous horizontal transfer of the *hyl* gene along with the genes responsible for high‐level gentamicin resistance.

The *efaA* gene plays a crucial role in the colonization and persistence of enterococcal infections, particularly in *E. faecalis* [83]. This gene was found in 91.2% of *E. faecalis* isolates studied. Several studies have reported a high prevalence of the *efaA* gene in this species [82, 83, 84]. We observed that all *efaA*‐positive isolates were biofilm producers, consistent with the findings of Yoon et al. [85].

In terms of *E. faecium* ‐specific virulence genes, *acm* and *fnm* were detected in 4 and 13 out of 17 *E. faecium* isolates, respectively. Previous studies have reported a higher frequency of *acm* [86, 87, 88], which plays a crucial role in binding to collagen and contributing to the development of endocarditis [89]. Furthermore, 11 out of 13 *fnm*‐positive isolates were associated with UTIs. According to the study by Freitas et al., *fnm* has a high prevalence among clinical and non‐clinical *E. faecium* isolates. It appears that genes such as *fnm*, which encode surface proteins, aid *Enterococcus* in effectively colonizing various biological and non‐biological surfaces. However, the expression of these genes may vary across different hosts [21].

We detected the *vanA* gene in 35.7% of the isolates, whereas the *vanB* gene was not observed in any of them, consistent with previous studies [10, 12]. The *vanB* gene is less prevalent than *vanA* in *Enterococcus* strains [19, 58, 90, 91, 92]. The occurrence of the *vanA* gene in VRE isolates was significant, and it was the main factor of vancomycin resistance. However, some isolates carrying the *vanA* gene remained susceptible to vancomycin, possibly due to a deficiency in the *vanA* operon [8]. Conversely, some of the *vanA*‐negative isolates exhibited resistance to vancomycin. Besides the *vanA* gene, resistance to vancomycin may be associated with other *van* genes such as *vanM*, *vanD*, and *vanE* [51]. In our study, vancomycin resistance and the presence of the *vanA* gene in *E. faecium* were significantly higher than in *E. faecalis* , consistent with previous studies [19, 45]. The literature indicates that *E. faecium* is the predominant species harboring the *vanA* gene in enterococci [51, 93].

Three AME‐encoding genes, *aph*(*3*′)*‐IIIa*, *ant*(*6*′)*‐Ia*, and *aac*(*6*′)*‐Ie‐aph*(*2*″)*‐Ia*, were the most prevalent in the isolates of the present study, whereas the *ant*(*4*′)*‐Ia*, *aph*(*2*″)*‐Ib*, *aph*(*2*″)*‐Ic*, and *aph*(*2*″)*‐Id* genes were not found among the isolates. Moreover, the occurrence of the *aac*(*6*′)*‐Ie‐aph*(*2*″)*‐Ia* gene in HLGR isolates and the existence of *aph*(*3*′)*‐IIIa*, *ant*(*6*′)*‐Ia*, and *aac*(*6*′)*‐Ie‐aph*(*2*″)*‐Ia* genes in HLSR isolates were statistically significant. According to several studies, the *aph*(*3*′)*‐IIIa*, *ant*(*6*′)*‐Ia*, and *aac*(*6*′)*‐Ie‐aph*(*2*″)*‐Ia* genes were the predominant aminoglycoside resistance genes, while the *ant*(*4*′)*‐Ia*, *aph*(*2*″)*‐Ib*, *aph*(*2*″)*‐Ic*, and *aph*(*2*″)*‐Id* genes were reported at low frequencies (0%–8%) [12, 19, 58, 94, 95]. The presence of these genes, along with horizontal gene transfer between enterococcal strains, contributes to the development of resistance and high‐level resistance to aminoglycosides. Considering the importance of combination therapy in treating life‐threatening enterococcal infections, the detection of high‐level aminoglycoside resistance (HLAR) is noteworthy [96].

In this study, only one isolate carried the *ant*(*3*″)*‐Ia* gene, found in *E. faecalis* from a wound infection. To the best of our knowledge, this is the first report of this gene in a clinical *Enterococcus* isolate in Iran. It was previously reported in the United States in 1999 [97] and in Portugal in 2023 (Accession number: OR879261). The *ant*(*3*″)*‐Ia* gene is responsible for high‐level resistance to streptomycin [98], and we detected it in an HLSR isolate.

## 5 Conclusion

The study highlights the substantial role of various virulence factors, biofilm formation, and antimicrobial resistance in the pathogenesis and severity of enterococcal infections. *E. faecalis* was identified as the most common species causing infections in hospitalized patients in Yazd, Iran. The frequency of HLGR and HLSR isolates, VRE, and resistance‐related genes was considerable. Furthermore, most of the isolates were MDR and biofilm producers, and the *gelE* gene was the most frequent virulence‐encoding gene. The notable prevalence of drug‐resistant isolates, along with the widespread presence of virulence genes, emphasizes the need for ongoing surveillance and the development of effective treatment policies to combat these infections.

## Conflicts of Interest

The authors declare no conflicts of interest.

## Data Availability

The authors confirm that the data supporting the findings of this study are available within the article.
